# Auraptene Enhances Junction Assembly in Cerebrovascular Endothelial Cells by Promoting Resilience to Mitochondrial Stress through Activation of Antioxidant Enzymes and mtUPR

**DOI:** 10.3390/antiox10030475

**Published:** 2021-03-17

**Authors:** Min Joung Lee, Yunseon Jang, Jiebo Zhu, Eunji Namgung, Dahyun Go, Changjun Seo, Xianshu Ju, Jianchen Cui, Yu Lim Lee, Hyoeun Kang, Hyeongseok Kim, Woosuk Chung, Jun Young Heo

**Affiliations:** 1 Department of Medical Science, Chungnam National University School of Medicine, Daejeon 35015, Korea; rmj1102@cnu.ac.kr (M.J.L.); yunseonj@cnu.ac.kr (Y.J.); zhujiebo2019@o.cnu.ac.kr (J.Z.); eunzie13@o.cnu.ac.kr (E.N.); goda1046@o.cnu.ac.kr (D.G.); justin20@o.cnu.ac.kr (C.S.); juxianshu1214@o.cnu.ac.kr (X.J.); 201860391@o.cnu.ac.kr (J.C.); leestar95@o.cnu.ac.kr (Y.L.L.); hyoeunKang@o.cnu.ac.kr (H.K.); hskim85@cnu.ac.kr (H.K.); 2 Department of Biochemistry, Chungnam National University School of Medicine, Daejeon 35015, Korea; 3 Infection Control Convergence Research Center, Chungnam National University School of Medicine, Daejeon 35015, Korea; 4 Department of Anesthesiology and Pain Medicine, Chungnam National University School of Medicine, Daejeon 35015, Korea; 5 Department of Anesthesiology and Pain Medicine, Chungnam National University Hospital, Daejeon 35015, Korea

**Keywords:** auraptene, endothelial cell, blood-brain barrier, antioxidant, mitochondria, mtUPR

## Abstract

Junctional proteins in cerebrovascular endothelial cells are essential for maintaining the barrier function of the blood-brain barrier (BBB), thus protecting the brain from the infiltration of pathogens. The present study showed that the potential therapeutic natural compound auraptene (AUR) enhances junction assembly in cerebrovascular endothelial cells by inducing antioxidant enzymes and the mitochondrial unfolded protein response (mtUPR). Treatment of mouse cerebrovascular endothelial cells with AUR enhanced the expression of junctional proteins, such as occludin, zonula occludens-1 (ZO-1) and vascular endothelial cadherin (VE-cadherin), by increasing the levels of mRNA encoding antioxidant enzymes. AUR treatment also resulted in the depolarization of mitochondrial membrane potential and activation of mtUPR. The ability of AUR to protect against ischemic conditions was further assessed using cells deprived of oxygen and glucose. Pretreatment of these cells with AUR protected against damage to junctional proteins, including occludin, claudin-5, ZO-1 and VE-cadherin, accompanied by a stress resilience response regulated by levels of *ATF5*, *LONP1* and *HSP60* mRNAs. Collectively, these results indicate that AUR promotes resilience against oxidative stress and improves junction assembly, suggesting that AUR may help maintain intact barriers in cerebrovascular endothelial cells.

## 1. Introduction

The blood-brain barrier (BBB) is a selectively permeable barrier that divides the central nervous system (CNS) from the peripheral circulation, preventing infectious substances and immune cells from entering the CNS [1]. Endothelial cells of the BBB interact with junctional proteins, such as occludin, claudin-5, zonula occludens-1 (ZO-1) and vascular endothelial cadherin (VE-cadherin), forming a barrier that prevents pathogens from crossing into the brain [2,3]. Loss of junctional proteins during pathological conditions, such as neurodegenerative diseases and multiple sclerosis, increases BBB permeability, allowing the infiltration of immune cells and resulting in brain damage [4,5,6]. Infection with severe acute respiratory syndrome coronavirus 2 (SARS-CoV-2) in patients with COVID-19 infection has been reported to cause neurological damage accompanying BBB disruption and inflammatory storm [7]. Maintenance of junctional proteins in the BBB is therefore needed to protect the CNS from infectious diseases.

Oxidative stressors such as reactive oxygen species (ROS) contribute to BBB disruption by damaging junctional proteins [8]. For example, elevated ROS levels following cerebral ischemic injury have been found to weaken the integrity of the BBB, resulting in BBB disruption [9]. Because endothelial cells are vulnerable to oxidative stress, and over 90% of intracellular ROS is produced by the mitochondrial respiratory chain, maintaining the integrity of the BBB requires strict regulation of mitochondrial respiration [10]. Therefore, the upregulation of antioxidant enzymes while maintaining mitochondrial function may preserve junctional proteins of endothelial cells in the BBB [11]. Although antioxidants are necessary to reduce ROS, the reactions involved can be strengthened by mitochondrial hormesis, which may constitute a therapeutic target to ameliorate diseases caused by oxidative stress [12]. Mild mitochondrial stress results in coordinated responses, such as the mitochondrial unfolded protein response (mtUPR), which can allow mitohormesis and stress resilience in cells [13]. Activation of the mtUPR can restore mitochondrial functions under stress conditions, such as increases in unfolded proteins and reductions in mitochondrial membrane potential [14,15].

Natural compounds, such as curcumin [16,17] and shizukahenriol [18], have been found to reduce oxidative stresses in the BBB microenvironment [19]. Few natural compounds, however, have been shown to enhance junctional proteins through the mtUPR. Auraptene (AUR) is a natural compound from citrus fruit [20] that was shown to decrease ROS by increasing antioxidant enzymes in neurotoxin-damaged dopaminergic neurons [21]. Although treatment with AUR increased antioxidant enzymes induced by phytohemagglutinin (PHA) stimulation of human lymphocytes [22], the effects of AUR on junctional proteins of endothelial cells that maintain BBB integrity have not yet been investigated. Moreover, the mechanisms of action by which AUR induces antioxidant activity in cells remain unclear. The present study analyzed the ability of AUR to maintain junctional proteins of cerebrovascular endothelial cells and its underlying mechanism of action. This study found that AUR treatment increased the expression of junctional proteins and upregulated the levels of mtUPR mRNA and of mRNAs encoding antioxidant enzymes. In addition, AUR pretreatment protected junctional protein disruption after oxygen-glucose deprivation, an in vitro condition similar to stroke. These findings suggest that AUR treatment can strengthen junctional proteins in cerebrovascular endothelial cells by inducing resilience to stress resulting from the induction of mtUPR.

## 2. Materials and Methods

### 2.1. Cell Culture and Oxygen-Glucose Deprivation (OGD)

bEnd.3 mouse cerebrovascular endothelial cell line (ATCC^®^ CRL2299™) was cultured in Dulbecco’s modified Eagle’s medium (DMEM) with 5% fetal bovine serum, 1% penicillin streptomycin at 37 °C and 5% CO_2_. Auraptene (AUR, 1 µM, Sigma-Aldrich, St. Louis, MO, USA) was pretreated for 24 h before oxygen-glucose deprivation. To model ischemia in vitro, confluent bEnd.3 cells were incubated with glucose-free DMEM in Billups-Rothenberg modular incubator chamber (Del Mar, San Diego, CA, USA) which was flushed for 5 min with 5% CO_2,_ 95% N_2_, and then sealed. The chamber was placed in at 37 °C incubator for 3 h.

### 2.2. Sulforhodamine B Assay (SRB) for Measurement of Cell Viability

bEnd.3 cells (5 × 10^3^ cells per well) were seeded in 96-well plates and incubated overnight. AUR 1, 2, 4 µM was treated for 24 h. After washing with PBS, cells were fixed with 10% TCA at 4 °C for 1 h. After washed and dried completely, the cells were stained with 0.4% SRB in 1% acetic acid for 20 min at room temperature. After washing 5 times with 1% acetic acid, the proteins were resolved with 10 mM unbuffered Tris (pH 10.5) Absorbance was read at 490 nm using a Multiskan Ascent plate reader (Thermo Scientific, Vantaa, Finland).

### 2.3. Extraction of Protein and Western Blot

Proteins of bEnd.3 cells pretreated with 1 µM AUR or vehicle and incubated in OGD condition or not were extracted using RIPA buffer with phosphatase inhibitor and protease inhibitor (Roche, Basel, Switzerland). 10 ug of proteins were loaded on SDS-PAGE gel and run by electrophoresis and then were transferred to PVDF membrane. After blocked with 3% BSA in TBST for 1 h at room temperature, the membranes were incubated at 4 °C, overnight with anti-occludin (Abcam, Cambridge, MA USA), anti-VE-cadherin (Abcam), anti-ZO-1 (Invitrogen, Waltham, MA, USA) and anti-β-actin (Santa Cruz Biotechnology, Santa Cruz CA, USA) antibody. After washing with TBS/T three times, the membranes were incubated with a secondary anti-rabbit or mouse IgG HRP antibodies (Pierce Biotechnology, Waltham, MA, USA) corresponded with the host of primary antibody for 2 h at room temperature. Protein bands were visualized by ECL system (Thermo Scientific) and Sensi-Q2000 Chemidoc (Lugen Sci, Buchen, Korea).

### 2.4. Isolation of RNA and Real-Time PCR

Total RNA was extracted using Trizol from bEnd.3 cells pretreated with 1 uM AUR or vehicle. cDNA was prepared from the total RNA with 5× RT premix (reverse transcription master premix). The mixtures of cDNA, SYBR Green PCR Master Mix (PhileKorea, Seoul, Republic of Korea) and primers were analyzed using Rotor Gene 6000 system (Corbett Life Science, Sydney, Australia). Used primers in this study: Antioxidant enzymes; NRF2 (forward 5′-CCAGAAGCCACACTGACAGA-3′, reverse 5′-GGAGAGGATGCTGCTGAAAG-3′), NQO1 (forward 5′-TTCTCTGGCCGATTCAGAGT-3′, reverse 5′-GGCTGCTTGGAGCAAAATAG-3′; GPX (forward 5′-GTCCACCGTGTATGCCTTCT-3′, reverse 5′-TCTGCAGATCGTTCATCTCG-3′), GST (forward 5′-GGCATCTGAAGCCTTTTGAG-3′, reverse 5′-GAGCCACATAGGCAGAGAGC-3′), Gclc (forward 5′-AGGCTCTCTGCACCATCACT-3′, reverse 5′-TGGCACATTGATGACAACCT-3′ (reverse); Gclm (forward 5′-TGGAGCAGCTGTATCAGTGG-3′, reverse 5′-GAGCAGTTCTTTCGGGTCA-3′), GR (forward 5′-CACGACCATGATTCCAGATG-3′, reverse 5′-CAGCATAGACGCCTTTGACA-3′), UPRmt; ATF4 (forward 5′-AGATGACCTGGAAACCATGC-3′, reverse 5′-GTGTCATCCAACGTGGTCAG-3′), ATF5 (forward 5′-ACCGCAAGCAAAAGAAGAGA-3′, reverse 5′-CAGCCTGGACCTGTACCCTA-3′), Lonp1 (forward 5′-GACAGAGAACCCGCTAGTGC-3′, reverse 5′-CTCAGTGGTTCTGGGATGGT-3′), Hspd1(forward 5′-GAGCTG GGTCCCTCACTCG-3′, reverse 5′-AGTCGAAGCATTTCTGCGGG-3′), Clpp (forward 5′-GCCATTCACTGCCCAATTCC-3′, reverse 5′-TGCTGACTCGATCACCTGTAG-3′), Chop (forward 5′-AACAGAGGTCACACGCACAT-3′, reverse 5′-ACTTTCCGCTCGTTCTCCTG-3′) and 18s rRNA (forward 5′-CGACCAAAGGAACCATAACT-3′, reverse 5′-CTGGTTGATCCTGCCAGTAG-3′).

### 2.5. Flow Cytometry

For analyzing mitochondrial membrane potential and ROS generation, the fluorescent dye, Tetramethylrhodamine, Ethyl Ester, Perchlorate (TMRE, Invitrogen), MitoSOX^TM^. Red reagent (Invitrogen) and CM-H_2_DCFDA (Invitrogen) were used. bEnd.3 cells were incubated with 1 µM AUR for 24 h. The media was removed and washed with HBSS/Ca/Mg and incubated for 30 min in the dark at 37 °C with final concentration 5 µM MitoSOX^TM^ or 5 µM CM-H_2_DCFDA or 100 nM TMRE in HBSS. Cells were washed with PBS and trypsinized. After the cells were suspended with PBS and avoided the light, FACS analysis was proceeded immediately by FACScan (BD Biosciences, Franklin Lakes, NJ, USA) using excitation/emission wavelengths of 485/535 nm for DCFDA and 510/580 nm for TMRE and MitoSOX^TM^, respectively. The values were expressed as mean fluorescence of the cell population.

### 2.6. Analysis of Oxygen Consumption Rate (OCR)

bEnd.3 cells (1 × 10^4^ cells per well) were incubated with 1 µM AUR for 24 h. The media were with 590 µL assay media in each well, the cell plate was incubated for 1 h at 37 °C in a non-CO_2_ incubation system. The XF24 biosensor cartridge was activated with 1 mL of XF24 calibrant solution (Agilent, Santa clara, CA, USA) per well for 24 h at 37 °C in a non-CO_2_ incubator. After measurement of basal OCR, 20 ug/mL oligomycin (an ATPase inhibitor, final conc. 2 μg/mL), 50 µM CCCP (an uncoupler, final conc. 5 µM) and 20 µM rotenone (mitochondrial complex I inhibitor, final conc. 2 µM) were injected into each well and sequentially measured three times OCR after injection of drug respectively at 37 °C.

### 2.7. Immunofluorescence Staining

bEnd.3 cells were seeded on coverslips coated with poly-d lysine and pretreated with 1 µM AUR or vehicle and incubated in OGD condition or not. After washed with warm PBS and fixed by 4% paraformaldehyde for 20 min, the cells were permeabilized with 0.1% TritonX-100 and 0.02% BSA in PBS for 30 min. And then the samples were blocked in 3% BSA with PBS for 30 min and incubated with following primary antibodies for 1.5 h overnight; anti-occludin (Invitrogen, Waltham, MA, USA), anti-VE-cadherin (Abcam), anti-ZO-1 (Invitrogen), anti-claudin-5 (Abcam) and anti-CD31 (Millipore, Burlington, MA, USA). The samples were washed with 0.2% BSA in PBS 3 times and incubated with the secondary antibodies for 1.5 h at room temperature; anti- mouse Alexa 488, anti-mouse Alexa 594, anti- rabbit Alexa 488, anti-rabbit Alexa 594 and anti-hamster Alexa 488-conjugated anti-IgG secondary antibodies (Jackson Laboratory, Bar Harbor, ME, USA). After incubated with DAPI and mounted with mounting medium (Dako North America Inc., Carpinteria, CA, USA), the slides were observed using Leica confocal microscope (Leica, Bensheim, Germany).

### 2.8. Statistical Analysis

All data are represented as mean values ± SEM. The statistical analysis of data was analyzed by two-tailed unpaired Student’s *t* test and one-way ANOVA test using Prizm version 8 (GraphPad software Inc., San Diego, CA, USA Statistical significance of all data was indicated by * *p* < 0.05, ** *p* < 0.01, or *** *p* < 0.001.

## 3. Results

### 3.1. AUR Treatment Increases Junctional Proteins in bEnd.3 Cells

**Figure 1:**
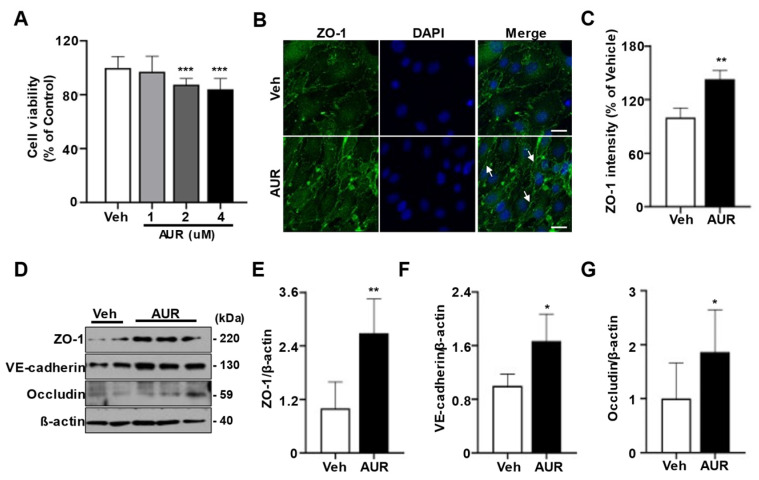
Effects of Auraptene (AUR) on junctional proteins expression in bEnd.3 cells. (**A**) bEnd.3 cells (5 × 10^3^ cells per well) seeded in 96-well plates were incubated in media containing 0, 1, 2, 4 µM of AUR for 24 h. Cell viability was measured by SRB assay. (**B**) bEnd.3 cells with vehicle (Veh) or 1 µM AUR were stained for ZO-1 (green) and DAPI (blue); Scale bar: 20 µm. Arrows indicate an increase of ZO-1 expression. (**C**) Relative ZO-1 intensity was quantified using ImageJ. (**D**) ZO-1, VE-cadherin and occludin expression were analyzed by Western blot after treatment of vehicle or 1 µM AUR for 24 h. (**E**–**G**) The protein levels of ZO-1, VE-cadherin and occludin were quantified using ImageJ. Data are presented as mean and ± SEM of three independent experiments (* *p* < 0.05, ** *p* < 0.01, *** *p* < 0.001 compared to Veh).

Oxidative stress can induce defects in tight junctions and adherens junctions of cerebrovascular endothelial cells [23]. Increasing ROS has been found to disrupt tight junctions between endothelial cells [10]. Because AUR induced antioxidant enzymes in response to a neurotoxic model of Parkinson’s disease [21,24], we investigated the effect of AUR on junction assembly of endothelial cells required for BBB maintenance. As shown using the sulforhodamine B (SRB) assay, incubation of bEnd.3 cells with >1 µM AUR for 24 h had a toxic effect on these cells; therefore, further experiments testing the physiological effectiveness of AUR were performed at a concentration of 1 µM (Figure 1A). To investigate the effect of AUR on junction assembly of bEnd.3 cells, cells were incubated with 1 µM AUR for 24 h and ZO-1 expression was evaluated by immunofluorescence staining. ZO-1 fluorescence expression was higher in cells treated with AUR than with vehicle (Veh; Figure 1B), with quantitative ZO-1 fluorescence intensity being 42% higher in the AUR than in the vehicle group (Figure 1C). In addition, the levels of the junctional proteins ZO-1, VE-cadherin, and occludin were 2.5-, 1.8- and 1.8-fold higher, respectively, in cells treated with AUR than with vehicle. Taken together, these results show that AUR treatment can enhance tight junctions and adherens junctions in cerebrovascular endothelial cells.

### 3.2. AUR Treatment Increases the Levels of mRNAs Encoding Antioxidant Enzymes in bEnd.3 Cells

**Figure 2:**
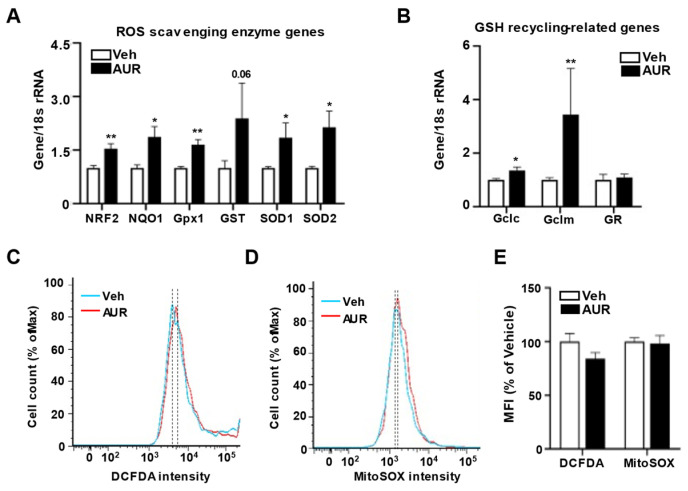
Increase of genes encoding antioxidant enzymes in bEnd.3 cells by AUR treatment. (**A**,**B**) mRNA expressions for ROS scavenging antioxidant enzymes and GSH recycling-related genes were analyzed using qPCR with bEnd.3 cells treated with vehicle or 1 µM AUR. (**C**,**D**) bEnd.3 cells were incubated with vehicle or 1 µM AUR. The cells were stained with 5 µM CM-H_2_DCFDA or 5 µM MitoSOX^TM^ and analyzed by flow cytometry. Total ROS was determined by DCFDA-stained cells (**C**) and mitochondrial ROS was determined by MitoSOX^TM^—Stained cells (**D**). Median fluorescence intensity (MFI) values are analyzed by FlowJo program. Data are presented as mean and ± SEM of three independent experiments (* *p* < 0.05, ** *p* < 0.01 compared to Veh).

Because AUR has antioxidant effects in dopaminergic neurons and human peripheral lymphocytes damaged by oxidative stress [21,24], we hypothesized that AUR could affect the levels of expression of antioxidant enzymes in cerebrovascular endothelial cells. Moreover, alterations in tight junctions were observed in intestinal epithelial cells, which play a role in barrier maintenance by increasing levels of antioxidant enzymes [25]. To investigate whether AUR-associated increases in junction protein expression were accompanied by increases in expression of antioxidant enzymes, we analyzed the effects of 1 µM AUR for 24 h on bEnd.3 cell levels on mRNAs encoding antioxidant enzymes, including ROS scavenging antioxidant enzymes such as *NRF2*, *NQO1*, *Gpx1*, *GST*, *SOD1* and *SOD2*; as well as GSH recycling-related genes, such as *Gclc*, *Gclm* and *GR*. The levels of mRNAs encoding superoxide dismutase (*SOD1*, *SOD2*, *GST*) and glutathione peroxidase (*Gpx1*) induced by NRF2 activation were more upregulated by AUR treatment than by vehicle (Figure 2A). Analysis of mRNAs related to GSH production and recycling found that AUR increased the levels of both *Gclc* and *Gclm* mRNAs, which encode enzymes that biosynthesize GSH, while not affecting the level of *GR* mRNA compared with cells treated with vehicle (Figure 2B).

These results suggested the need to examine the levels of ROS regulated by antioxidant enzymes. ROS levels in bEnd.3 cells treated with 1 µM AUR for 24 h were determined using DCFDA fluorescent dye, an indicator of cytosolic ROS, and MitoSOX^TM^ red reagent, an indicator of mitochondrial superoxide, followed by flow cytometry (Figure 2C,D). The DCFDA intensity tended to be lower in the AUR than in the vehicle group, although the difference was not statistically significant. MitoSOX^TM^ intensity also did not differ between the vehicle and AUR groups (Figure 2E). Taken together, these results suggest that AUR treatment enhances the level of transcription of genes encoding antioxidant enzymes, but does not alter intracellular ROS levels once no extracellular insult occurred.

### 3.3. AUR Reduces Mitochondrial Membrane Potential Maintaining Mitochondrial Respiration

**Figure 3:**
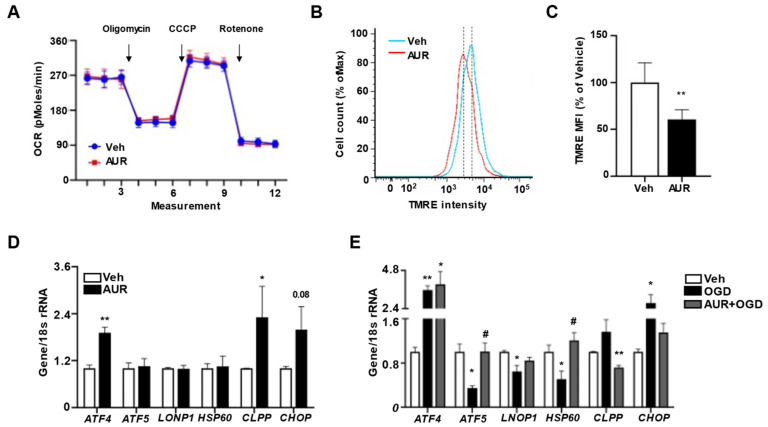
Loss of mitochondrial membrane potential accompanying induction of mtUPR in bEnd.3 cells by AUR treatment. (**A**) Oxygen consumption rate (OCR) was measured in bEnd.3 cells treated with vehicle or 1 µM AUR for 24 h. (**B**) bEnd.3 cells were incubated with vehicle or 1 µM AUR. The cells were stained with 100 nM TMRE and analyzed by flow cytometry. (**C**) Mitochondrial membrane potential was determined by TMRE-stained cells. The Median fluorescence intensity (MFI) values are analyzed by FlowJo program. (**D**) Expression of mRNA for mitochondrial unfolded protein response (mtUPR) genes was examined after 1 µM AUR treatment using qPCR. (**E**) Expression of mRNA for mitochondrial unfolded protein response (mtUPR) genes was examined with bEnd.3 cells incubated in oxygen-glucose deprivation (OGD) condition for 3 h after pretreated vehicle or 1 µM AUR 24 h. Data are presented as mean and ± SEM of three independent experiments (* *p* < 0.05, ** *p* < 0.01 compared to Veh, # *p* < 0.05 compared to OGD).

Our previous finding, that AUR can enhance mitochondrial respiration in dopaminergic neurons suppressed by neurotoxin [21], suggested the need to investigate mitochondrial respiration in bEnd.3 cells by measuring oxygen consumption rate (OCR). Incubation of bEnd.3 cells with 1 µM AUR for 24 h did not affect basal OCR (Figure 3A), nor did it alter ATP production following oligomycin injection, maximal respiration measured after CCCP, or non-mitochondrial OCR measured after rotenone treatment. The extracellular acidification rate (ECAR) was also similar in the two groups (Figure S1). These results showed that mitochondrial function was maintained during situations in which levels of antioxidant enzymes increased.

AUR was found to dose-dependently reduce mitochondrial membrane potential in human gastric cancer cells [26]. Because mitochondrial stress has been reported to alter mitochondrial membrane potential [27], and antioxidant defense mechanisms to maintain mitochondrial homeostasis by modulating mitochondrial membrane potential and ATP production [28], we hypothesized that AUR can affect mitochondrial membrane potential, resulting in mild stress for induction of antioxidant enzymes. Mitochondrial membrane potential was analyzed in bEnd.3 cells treated with 1 µM AUR for 24 h by flow cytometry using TMRE fluorescence dye (Figure 3B). As expected, mitochondrial membrane potential in AUR treated cells was 69.3% of that in vehicle-treated cells (Figure 3C). Previous studies suggest that slight alteration of mitochondrial membrane potential can allow recruitment and assembly of signaling molecules that restore mitochondrial homeostasis which protect mitochondrial dysfunction, while more severe alterations in mitochondrial membrane potential can induce cell death accompanied with an increase of ROS [13]. It is possible that AUR-induced reduction in mitochondrial membrane potential activated a mitochondrial homeostasis signaling pathway, which prevented the reduction in OCR.

### 3.4. AUR Promotes Resilience to Stress by Induction of mtUPR in bEnd.3 Cells

Low mitochondrial membrane potential can activate the mtUPR, which helps cells adjust to stress conditions by restoring mitochondrial function [12,13]. Activation of this response can restore mitochondrial function under stress conditions, during which misfolded proteins accumulate within mitochondrial matrices, with cells becoming resilient to stress [14]. Furthermore, mtUPR activation can induce a mitochondrial protective reaction such as antioxidant activity [29]. Therefore, we investigated the effects of AUR on mRNAs levels of mtUPR-related genes activated under mitochondrial stress conditions to maintain mitochondrial function. AUR treatment of bEnd.3 cells increased the levels of *ATF4*, *CLPP* and *CHOP* mRNAs, but did not affect the levels of *ATF5*, *LONP1* and *HSP60* mRNAs (Figure 3D). To test whether mtUPR is affected under oxidative stress conditions and whether AUR pretreatment alters the mtUPR, we analyzed the effects of pretreatment with 1 µM AUR for 24 h on mRNA levels of mtUPR-related genes in bEnd.3 cells incubated under OGD conditions for 3 h, an in vitro mimic of stroke [30]. Surprisingly, the levels of *ATF5*, *LONP1* and *HSP60* mRNAs, which were increased in response to the accumulation of unfolded proteins in mitochondria, were decreased by OGD, whereas expression of these genes was unaffected by AUR pretreatment (Figure 3E). Such differences in mtUPR expression may be due to differences in the severity of stress [14]. In fact, our results show that OGD significantly reduced cell viability compared to vehicle group, representing the severity of stress caused by OGD (Supplementary Figure S1B). OGD upregulated the expression of *ATF4* mRNA, but this upregulation was unaffected by pretreatment with AUR. OGD also increased the levels of *CLPP* and *CHOP* mRNAs, but this increase was abrogated by AUR pretreatment. These findings suggest that AUR induction of mild mitochondrial stress, resulting in mitochondrial membrane depolarization, leads to the induction of mtUPR expression and that the latter can contribute to resilience to oxidative stress in bEnd.3 cells.

### 3.5. Pretreatment with AUR Protects against OGD Reduction in Junctional Protein Expression

**Figure 4:**
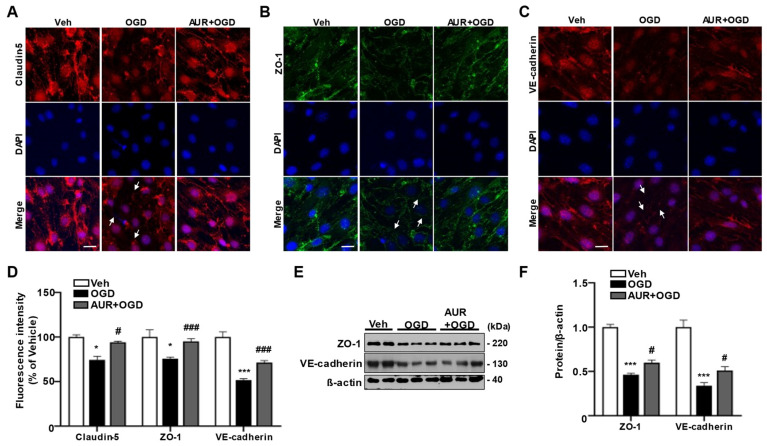
Protective effects of AUR pretreatment on the reduction of junctional protein by OGD in bEnd.3 cells.bEnd.3 cells were incubated in OGD condition for 3 h after treated with vehicle or 1 µM AUR 24 h. bEnd.3 cells were stained with claudin-5 (red) (**A**), ZO-1 (green) (**B**) and VE-cadherin (red) (**C**) with DAPI (blue); Scale bar: 20 µm. Arrows indicate junctional protein disruption. (**D**) Relative fluorescence intensity of markers was quantified using ImageJ. (**E**) ZO-1 and VE-cadherin expression were analyzed by Western blot with bEnd.3 cells incubated in OGD condition for 3 h after treatment of vehicle or 1 µM AUR for 24 h. Data are presented as mean and ± SEM of three independent experiments (* *p* < 0.05, *** *p* < 0.001 compared to Veh, # *p* < 0.05, ### *p* < 0.001 compared to OGD).

To investigate whether AUR induction of the mtUPR protects against OGD-induced damage to junctional proteins in bEnd.3 cells, we assessed the expression of the junctional proteins claudin-5, ZO-1 and VE-cadherin using immunofluorescence staining. bEnd.3 cells were pretreated with 1 µM AUR or vehicle for 24 h and subsequently incubated under OGD conditions for 3 h (Figure 4A–C). OGD reduced the fluorescence intensities of claudin-5, ZO-1 and VE-cadherin by 74.2%, 75.4% and 51.5%, respectively, compared with vehicle (Figure 4D). These reductions in junctional protein expression were prevented by AUR pretreatment. Specifically, pretreatment with AUR increased the fluorescence intensities of claudin-5, ZO-1 and VE-cadherin by 18.1%, 18% and 20%, respectively, compared with OGD alone (Figure 4D). The levels of ZO-1 and VE-cadherin proteins were 50% lower in cells subjected to OGD conditions than in cells exposed to vehicle alone, but these reductions were prevented by pretreatment with 1 µM AUR for 24 h (Figure 4E,F). In addition, increases in ROS level and antioxidant enzyme expression via OGD were reduced by AUR pretreatment (Supplementary Figure S2). We further observed that AUR pretreatment prevented the significant decrease in mitochondrial membrane potential due to OGD (Supplementary Figure S2).

**Figure 5:**
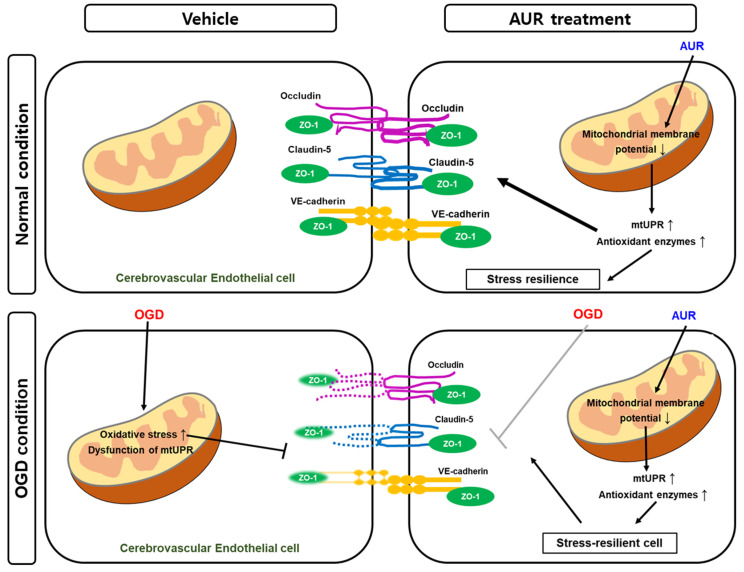
Schematic representation of the effect of AUR in normal condition and the protective mechanism in vitro ischemic injury model. AUR treatment can enhance junction assembly and induce resilience to oxidative stress in cerebrovascular endothelial cells by altering levels of mtUPR and antioxidant enzymes, resulting in low mitochondrial membrane potential. This reaction contributes mitochondrial stress resilience and it exhibits by alleviating degradation of junctional proteins when the cerebrovascular endothelial cells were put in OGD condition, in vitro ischemic injury model.

Taken together, these results suggest that AUR protects bEnd.3 cells from the OGD-induced decrease in junctional proteins by enhancing resilience to mitochondrial stress through the induction of mtUPR. Thus, AUR induces antioxidant enzymes and alters mtUPR expression through mild depolarization of mitochondrial membrane potential (Figure 5). These effects improved junctional protein expression in cerebrovascular endothelial cells as well as contributing to their resilience to oxidative stress and OGD-induced damage to junctional proteins.

## 4. Discussion

BBB disruption is observed in neurodegenerative diseases and infectious diseases such as COVID-19 infection [3,5]. The selective permeability of the BBB results from junction assembly, including tight junctions and adherens junctions of cerebrovascular endothelial cells [2]. BBB integrity is maintained by the preservation of junctional proteins in endothelial cells. The present study demonstrated that AUR enhances the expression of junctional proteins and protects against damage to the junctional assembly induced by ischemic conditions in bEnd.3 cells.

The junctional complexes of endothelial cells in the tight junctions of the BBB include occludin, claudin-5 and other junction-associated proteins, as well as ZO-1, which regulates vascular permeability [31]. Because occludin and claudin-5 are key structural components of the BBB, their dysfunction can cause BBB breakdown [32]. ZO-1 stabilizes junctional complexes by interacting with occludin and claudin-5 [33]. The adherens junction protein VE-cadherin is also involved in the control of vascular barrier function [34]. AUR treatment increased occludin, ZO-1 and VE-cadherin expression, as well as protecting these proteins, along with claudin-5, from the effects of OGD conditions. Disruption of these junctional complexes of the BBB is a hallmark of many CNS diseases, including stroke, neurodegenerative diseases and multiple sclerosis [35]. Our finding, that AUR protects junctional assemblies from the effects of ischemic conditions, suggests that AUR may also be applicable to patients with stroke, neurodegenerative diseases and multiple sclerosis, which cause neuroinflammation by BBB disruption. Moreover, we previously reported that alterations in the actin cytoskeleton of endothelial cells caused by dysfunctional mitochondrial respiration disrupted tight junctions [36]. Because regulation of the actin cytoskeleton is key to maintaining junction complexes, further studies are needed to investigate the effects of AUR on dysregulation of the actin cytoskeleton caused by mitochondrial dysfunction.

ROS have been found to increase the permeability of the BBB by altering tight junctions, suggesting that therapeutic targeting of the regulation of antioxidant activity can preserve the integrity of the BBB [37,38]. The relationship between antioxidant enzymes and junction expression has also been observed in intestinal epithelial cells, which have barrier functions similar to that of the BBB. Spermine, a polyamine, increases the levels of ZO-1 and occludin mRNAs in the ileum, accompanied by increases in the expression of antioxidant enzymes, such as GST, Gpx1 and NRF2 [39]. In addition, exercise-induced upregulation of SOD1 and SOD2 expression was accompanied by an increase in expression of the tight junction gene zonulin in rat ileum [25]. Taken together with our findings, these results suggest that upregulation of antioxidant enzymes can enhance expression of junctional proteins. Although AUR was shown to induce NRF2 expression, followed by increases in the levels of mRNAs encoding antioxidant enzymes [21], the mechanisms underlying the AUR-induced changes in antioxidant enzyme expression remain unclear and require further investigation.

One indirect cause of changes in expression of antioxidant enzymes may be the induction of mtUPR, a stress defense mechanism involving alterations in mitochondrial membrane potential. Because reduced mitochondrial membrane potential has been reported to trigger mtUPR induction [15], we assessed the effect of AUR on mitochondrial membrane potential, finding that AUR treatment reduced mitochondrial membrane potential. Mild uncoupling not inducing a dysfunction of mitochondrial respiration can alleviate pathologic conditions associated with oxidative stress [40] and mildly depolarized mitochondrial membrane potential can have anti-aging effects [41]. A low concentration of the decoupler 2,4-dinitrophenol-protected neurons from amyloid-β aggregation [42], and the novel cationic mitochondrial uncoupler C4R1 reduced fat mass in a mouse model of obesity [43]. mtUPR is typically activated for survival against stress, a biological response defined as mitohormesis [13]. Mild stress can activate mtUPR/mitohormesis which leads to stress resilience in cells, and has been suggested as a novel therapeutic strategy [44]. Our results suggest that AUR pretreatment activates mtUPR by causing mild stress (lower mitochondrial membrane potential). In contrast, severely stressful conditions such as OGD (an in vitro ischemic model which induces ROS [45]) can reduce mtUPR gene expression and eventually cause cell death. However, cells that have undergone stress resilience through mtUPR activation by AUR pretreatment may survive. We found that AUR induction or increase of mtUPR promoted resilience to stress by regulating *ATF5*, *LONP1* and *HSP60* mRNA expression. Moreover, by analyzing protein levels using immunofluorescence staining and western blotting, we also demonstrated that AUR protected bEnd.3 cells against OGD-induced reductions of junctional proteins. Although these findings suggested that AUR indirectly increases antioxidant enzymes by increasing mtUPR levels through increased stress resilience, further research on specific pathways associated with alterations in mtUPR levels is required.

To our knowledge, these results are the first to suggest that AUR can induce resilience to oxidative stress in cerebrovascular endothelial cells by altering levels of mtUPR and antioxidant enzymes, resulting in low mitochondrial membrane potential. In addition, these findings suggest that AUR enhances the expression of junctional proteins in cerebrovascular endothelial cells and that this natural compound may be effective in maintaining barrier function in endothelial cells of the BBB.

## 5. Conclusions

Maintenance of junctional proteins in the BBB is needed to protect the CNS from infectious agents. Oxidative stress is associated with BBB disruption through the dysregulation of junctional proteins in cerebrovascular endothelial cells. Few natural compounds, however, have been found to modulate antioxidant enzymes while enhancing cellular resilience to oxidative stress. AUR can enhance junctional protein expression and induce the expression of antioxidant enzymes and resilience to oxidative stress. The present study suggests that AUR may be a natural therapeutic compound that maintains integrity of the BBB.

## Data Availability

Data are included in the article.

## Supplementary Materials

Supplementary materials can be found at https://www.mdpi.com/2076-3921/10/3/475/s1.
