# Supplementary material for: Auraptene Enhances Junction Assembly in Cerebrovascular Endothelial Cells by Promoting Resilience to Mitochondrial Stress through Activation of Antioxidant Enzymes and mtUPR
Source: Antioxidants (Basel). 2021 Mar 17;10(3):475. doi: 10.3390/antiox10030475 (PMC8002628; doi:10.3390/antiox10030475)
Supplement: Supplementary file 1 [file antioxidants-10-00475-s001.pdf]

Auraptene Enhances Junction Assembly in Cerebrovascular Endothelial Cells by Promoting Resilience to Mitochondrial Stress through Activation of Antioxidant Enzymes and mtUPR

Min Joung Lee [1,2,3], Yunseon Jang [1,2,3], Jiebo Zhu [1,2,3], Eunji Namgung [1,2,3], Dahyun Go [1,2,3], Changjun Seo [1,2,3], Xianshu Ju [1,3], Jianchen Cui [1,3], Yu Lim Lee [1,3], Hyoeun Kang [1,2], Hyeongseok Kim [1,2], Woosuk Chung [1,4,5,*] and Jun Young Heo [1,2,3,*]#

1 Department of Medical Science, Chungnam National University School of Medicine, Daejeon 35015, Korea; rmj1102@cnu.ac.kr (M.J.L.); yunseonj@cnu.ac.kr (Y.J.); zhujiebo2019@o.cnu.ac.kr (J.Z.); eunzie13@o.cnu.ac.kr (E.N.); goda1046@o.cnu.ac.kr (D.G.); justin20@o.cnu.ac.kr (C.S.); hyoeunKang@o.cnu.ac.kr (H.K.); juxianshu1214@o.cnu.ac.kr (X.J.); 201860391@o.cnu.ac.kr (J.C.); leestar95@o.cnu.ac.kr (Y.L.L.); hskim85@cnu.ac.kr (H.K.)

2 Department of Biochemistry, Chungnam National University School of Medicine, Daejeon 35015, Korea

3 Infection Control Convergence Research Center, Chungnam National University School of Medicine, Daejeon 35015, Korea

4 Department of Anesthesiology and Pain Medicine, Chungnam National University School of Medicine, Daejeon 35015, Korea

5 Department of Anesthesiology and Pain Medicine, Chungnam National University Hospital, Daejeon 35015, Korea

* Correspondence: woosuk119@cnu.ac.kr (W.C.); jyheo@cnu.ac.kr (J.Y.H.); Tel.: +82-042-580-8222 (J.Y.H.)

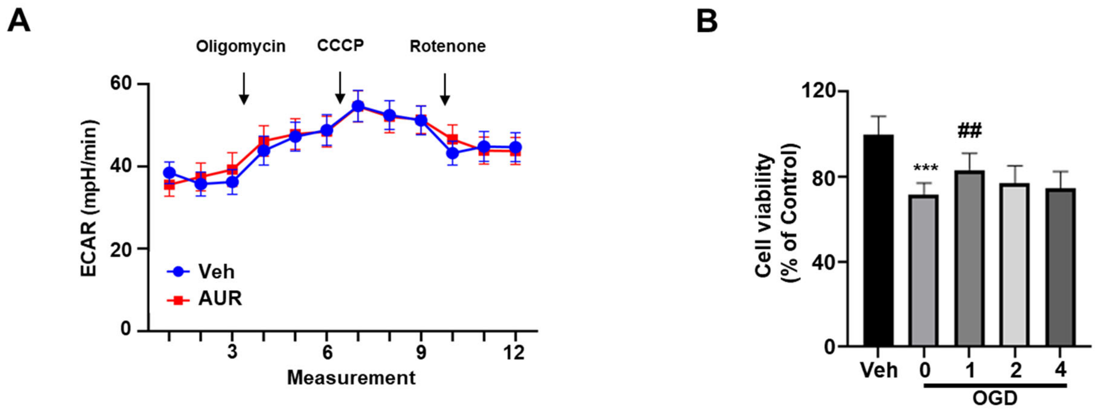

Supplementary Figure 1.

(A) extracellular acidification rate (ECAR) was measured in bEnd.3 cells treated with vehicle or 1 μM AUR for 24h.

(B) bEnd.3 cells (5 x 10^3 cells per well) seeded in 96-well plates were incubated in OGD condition for 3h after incubated in media containing 0, 1, 2, 4 μM of AUR for 18h. Vehicle group was

incubated in normal condition for 24h. Cell viability was measured by SRB assay (*** $p < 0.001$ compared to Veh, # $p < 0.05$ compared to OGD).

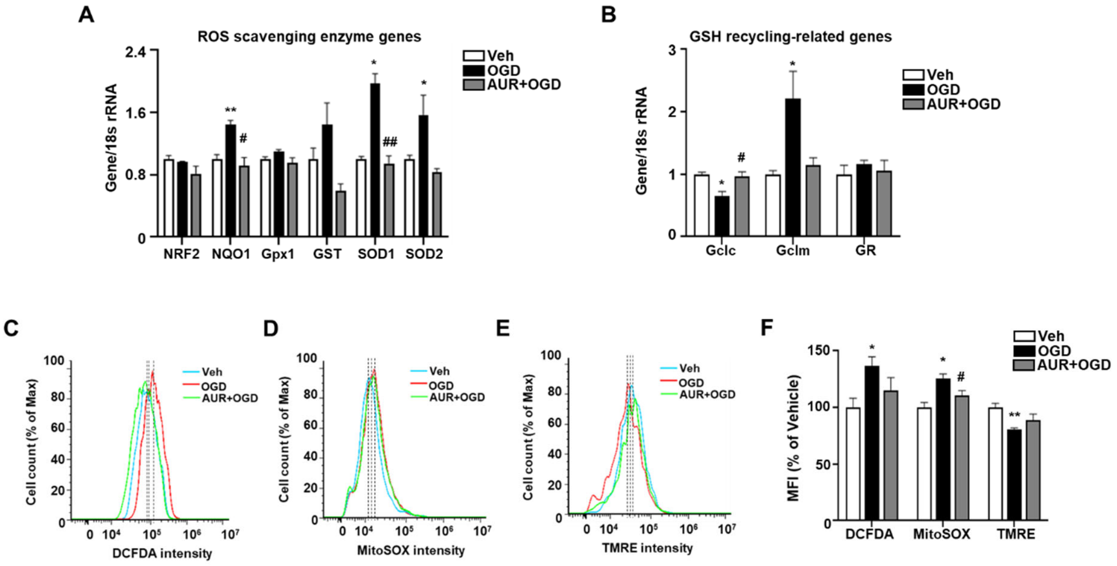

Supplementary Figure 2.

(A, B) mRNA expressions for ROS scavenging antioxidant enzymes and GSH recycling-related genes were analyzed using qPCR with bEnd.3 cells incubated in OGD 3h after treatment of vehicle or 1 μM AUR.

(C, D) bEnd.3 cells were incubated in OGD 3h after treatment of vehicle or 1 μM AUR. The cells were stained with 5 μM CM-H_2DCFDA or 5 μM MitoSOX™ or 100 nM TMRE and analyzed by flow cytometry. Total ROS was determined by DCFDA-stained cells(C), mitochondrial ROS was determined by MitoSOX™ – stained cells (D) and mitochondrial membrane potential was determined by TMRE-stained cells (E). (F) Median fluorescence intensity (MFI) values are analyzed by FlowJo program. Data are presented as mean and ± SEM of three independent experiments (* $p < 0.05$, ** $p < 0.01$ compared to Veh, # $p < 0.05$ compared to OGD).

.
